# In Vitro Evaluation of Sulforaphane and a Natural Analog as Potent Inducers of 5-Fluorouracil Anticancer Activity

**DOI:** 10.3390/molecules23113040

**Published:** 2018-11-21

**Authors:** Małgorzata Milczarek, Lidia Mielczarek, Katarzyna Lubelska, Aleksandra Dąbrowska, Zdzisław Chilmonczyk, Dariusz Matosiuk, Katarzyna Wiktorska

**Affiliations:** 1 Department of Drug Biotechnology and Bioinformatics, National Medicines Institute, 30/34 Chełmska St, 00-725 Warsaw, Poland; m.milczarek@nil.gov.pl (M.M.); l.mielczarek@nil.gov.pl (L.M.); k.lubelska@nil.gov.pl (K.L.); a.dabrowska@nil.gov.pl (A.D.); z.chilmonczyk@nil.gov.pl (Z.C.); 2 Chair and Department of Synthesis and Chemical Technology of Pharmaceutical Substances, Medical University of Lublin, 1 Aleje Racławickie St, 20-059 Lublin, Poland; dariusz.matosiuk@umlub.pl

**Keywords:** sulforaphane, 5-fluorouracil, drug interactions, apoptosis, cell cycle

## Abstract

Isothiocyanates (R-NCS) are sulphur-containing phytochemicals. The main source are plants of the *Brassicaceae* family. The best known plant-derived isothiocyanate is sulforaphane that has exhibited anticancer activity in both in vivo and in vitro studies. Recent attempts to expand their use in cancer therapy involve combining them with standard chemotherapeutics in order to increase their therapeutic efficacy. The aim of this paper is to determine the impact of sulforaphane and its natural analog alyssin on the anticancer activity of the well-known anticancer drug 5-fluorouracil. The type of drug-drug interactions was determined in prostate and colon cancer cell lines. Confocal microscopy, western blot and flow cytometry methods were employed to determine the mechanism of cytotoxic and cytostatic action of the combinations. The study revealed that additive or synergistic interactions were observed between 5-fluorouracil and both isothiocyanates, which enhanced the anticancer activity of 5-fluorouracil, particularly in colon cancer cell lines. An increased cytostatic effect was observed in case of alyssin while for sulforaphane the synergistic interaction with 5-fluorouracil involved an intensification of apoptotic cell death.

## 1. Introduction

In 2015 one out of six deaths was caused by malignant neoplasms, according to the WHO. Apart from lung, stomach and skin cancers, the neoplasms most frequently observed in men are those of the large intestine and prostate [1].

5-Fluorouracil (5-FU), commonly used to treat solid tumors of e.g., digestive system or breast, is one of the most extensively used drugs in anticancer therapy. Like any other anticancer drug, 5-FU has side effects (e.g., myelosuppression, cardiotoxicity) which in many cases severely limit treatment options. The search of new, especially more effective and less toxic, therapeutic combinations or new drugs seems of great importance in this respect [2].

Combinations of conventional chemotherapy with natural compounds of documented (in vitro) anticancer activity, e.g., resveratrol or curcumin, etc. have become one of the concepts in the search for new ways to treat cancer [3,4,5]. Most of these compounds are already part of the human diet, so they are assimilated well and, most importantly, as shown in clinical trials, demonstrate low toxicity. Isothiocyanates (ITCs), present in plants of the *Brassicaceae* family, are one of the groups of compounds possessing this activity.

Sulforaphane is by far the most extensively characterized ITC, with influence described at every stage of carcinogenesis, from the initiation phase, where it stimulates deactivation and removal of carcinogens, to the promotion and progression phase, where it induces cancer cells’ death, reduces their proliferation, blocks metastasis and angiogenesis [6,7,8]. Furthermore, in vitro studies showed that sulforaphane may interact with other drugs conventionally used in oncology, e.g., doxorubicin, oxaliplatin, bortezomib, gemcitabine in salivary gland (ACC-M and ACC-2), colon (CaCo-2), ovarian (A270), and breast (MCF-7) cancer cell lines [9,10,11]. Induction of apoptosis lies behind the mechanism of this synergistic interaction in most cases. At the same time, our team indicated on a model of normal cells, untransformed Chinese hamster fibroblasts, that sulforaphane did not enhance the cytotoxicity or the cytostatic activity of 5-FU [12].

**Figure 1:**
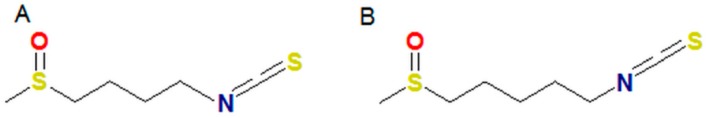
Structural formulas of isothiocyanates: (**A**) sulforaphane; (**B**) alyssin.

Like sulforaphane, alyssin, also present in plants of the *Brassicaceae* family, is widespread in *Alyssum* sp. As regards its structure, alyssin differs from sulforaphane only in having its methylene chain extended by one –CH_2_– group (Figure 1). Kim et al. indicated that the antineoplastic activity of alyssin against four colon cancer cell lines: Caco-2, HT-29, LoVo, and HCT116 was slightly weaker than that of sulforaphane [13]. By contrast, studies of Cierpiał at al. showed that such a structure modification stimulates the antineoplastic activity of ITCs [14]. Alyssin is more cytotoxic and selective against neoplastic cells than sulforaphane, as it was shown by Misiewicz at al. on a leukemia cell line (CCRF-SB) and on lymphoblastoids. Due to its selective influence on detoxifying enzymes only in untransformed lymphocytes, this group of researchers also characterized alyssin as a potential adjuvant in the treatment of leukemias [15]. Lubelska et al., on the other hand, in a Caco-2 colon cell line showed that at non-cytotoxic concentrations and after being administered with non-oncological drugs the compound affected enzymes taking part in detoxification to a greater degree than sulforaphane [16,17].

Therefore, in this paper we investigated the anticancer properties of the combinations of 5-FU with sulforaphane and alyssin. The research was conducted on four cell lines: colon cancer: HT-29, Caco-2, and prostate cancer: PC-3, LNCaP. In this study we show that thanks to strong cytostatic activity which subsequently induces cytotoxic effects, the combination of sulforaphane and 5-FU proved to be the most effective one.

## 2. Results and Discussion

Still one of the most commonly used chemotherapeutics, 5-FU causes severe side effects. Therefore, researchers are trying to find new therapy schemes to reduce patients’ risk of toxicity and enhance the drug’s effectiveness. In this paper we propose a new combination based on 5-FU and the naturally occurring ITCs sulforaphane and alyssin, which anticancer properties have been well characterized. As a result of using combinations of compounds, in comparison to their separate administration, beneficial interactions may occur. Their occurrence enhances anticancer effect and, consequently, makes it possible to reduce the dosage of the compound.

### 2.1. Type of Interaction between ITC and 5-FU

**Figure 2:**
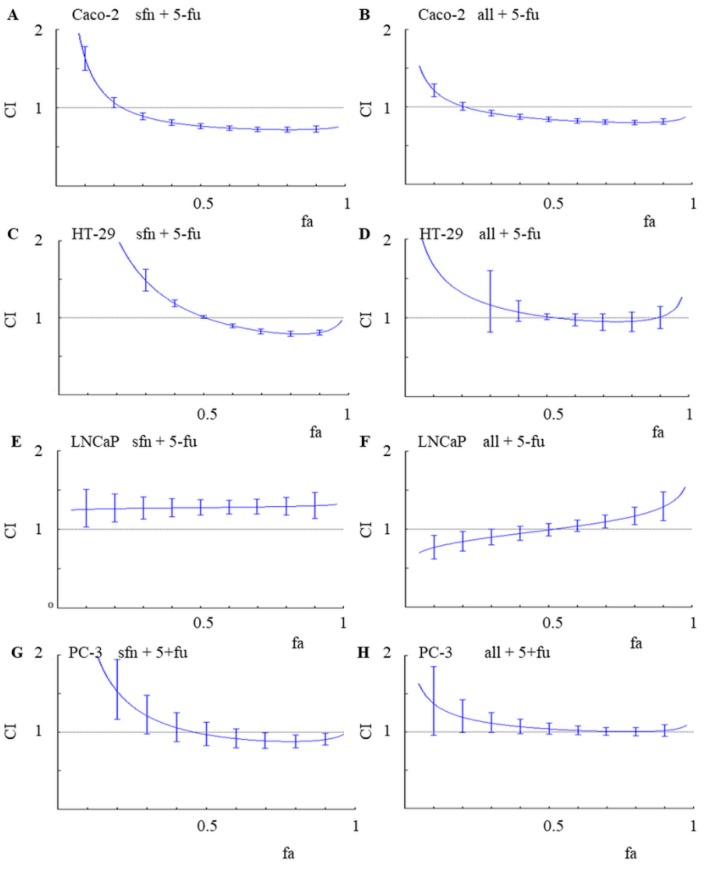
Combination index values (CI) depending on the fraction affected (fa) in Caco-2 (**A**,**B**), HT-29 (**C**,**D**) colon cancer cell lines and LNCaP (**E**,**F**) and PC-3 (**G**,**H**) prostate cancer cell lines, for 5-FU administrations with sulforaphane (**A**,**C**,**E**,**G**) and 5-FU administrations with alyssin (**B**,**D**,**F**,**H**). CI > 1—antagonism, CI < 1—synergism, CI ± 1.0—an additive effect. The Chou-Talalay’s method was used to determine CI.

The tests were carried out on two cancer types, two colon cancer cell lines (fast and slowly proliferating) and two prostate cancer cell lines (hormone resistant and hormone sensitive). The Chou-Talalay method was used to determine the type of interaction with respect to anticancer activity [18]. Three types of interactions were distinguished on the basis of a combination index (CI) value: synergism (CI < 1.1), antagonism (CI > 1.1) and an additive effect (CI (0.9–1.1)). Figure 2 shows CI values in colon and prostate cancer lines, depending on the percentage (fraction) of cells which growth was affected (fa).

Among the evaluated combinations, a significantly higher anticancer activity of the 5-FU and ITC combinations was observed in cancer colon cells. Synergism was observed in the Caco-2 colon cancer line for both combinations: sulforaphane and 5-FU as well as alyssin and 5-FU, reaching the CI values of about 0.7 at fa ≥ 0.5. Synergism means that the effect of the combination is stronger than if its two compounds where to be administered separately. In HT-29, the second tested colon cancer line, synergism was observed in the combination of sulforaphane and 5-FU, with CI about 0.8 for fa values ranging from 0.7 to 0.9. An additive effect (CI value about 1.0) was observed in HT-29 cells treated with the combination of alyssin and 5-FU.

An additive effect (CI value about 1.0) was observed also in PC-3 cells treated with both combinations, with fa > 0.5. In contrast, antagonism was primarily noted in the LNCaP cell line. For example, irrespectively of fa, CI value was about 1.3 in the cells treated with the combination of sulforaphane and 5-FU. In comparison with an additive model of action, antagonism reduces the overall therapeutic effect of the combination of agents, according to the Chou-Talalay method. This understandably renders antagonistic interactions highly undesirable in any search for new successful anticancer treatments [18].

**Figure 3:**
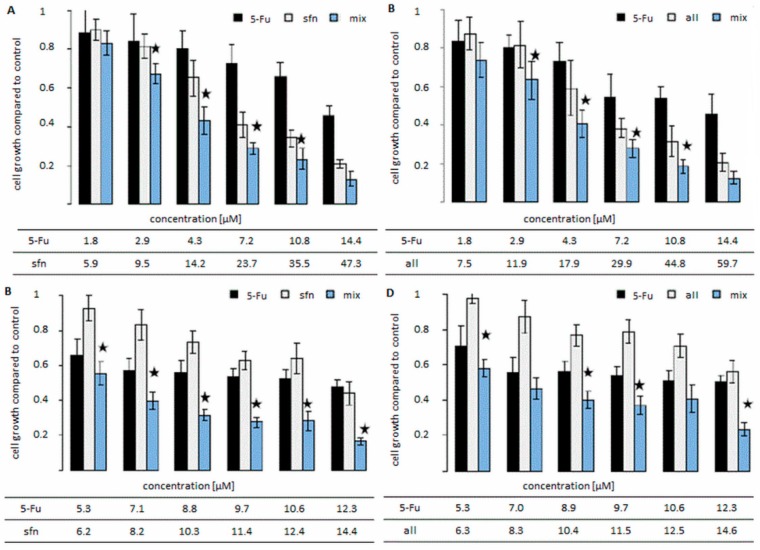
The changes in cell growth of colon cancer cell lines after the administration of compounds alone and in combination: 5-fluorouracil (5-FU) with: sulforaphane (sfn) in Caco-2 (**A**), andHT-29 (**C**) cell lines and alyssin (all) in Caco-2 (**B**), and HT-29 (**D**) cell lines. * The cell growth values after the administration of combination statistically significantly different from the cell growth value after singular administrations of ITC and 5-FU, *p* < 0.05. The cell growth was determined by MTT method. The combination: cells were incubated with ITC for 24 h and then with 5-FU for 72 h. In singular administrations one component of the combination was used.

As determined by us, the type of interaction correlated with the changes observed in the cell growth data (Figure 3). The growth of the cells in colon cancer cell lines was mostly decreased after the administration of the combination, in comparison to the growth recorded after administering only one agent. The combination of sulforaphane and 5-FU in HT-29 cell line led to the greatest decrease in cell growth. In this line the decrease in most cases reached 50% after administering the compounds in a combination, in comparison to their separate administration.

This is the first publication on the interaction of alyssin with an anticancer drug on an in vitro model. Śliwka et al. had examined the interaction of Selol, an organic compound of anti-cancerous properties containing Se^4+^, with alyssin and sulforaphane and its analogues. Those researchers’ results, like ours, revealed a possible additive effect or antagonistic interaction of alyssin. Likewise, it was confirmed that the type of interaction between sulforaphane and the anticancer compound was more synergistic than combinations with alyssin [19]. These results suggest that the elongation of the sulforaphane carbon chain weakened the beneficial molecular interaction with anticancer compounds.

The Chou-Talalay method enabled us to determine the dose-reduction index (DRI) value. The DRI determines how much, under the constraint of providing the same therapeutic effect, the concentration of compounds can be reduced as a result of being administered in combination in comparison to separate administration. The DRI calculated for 5-FU and ITC in colon cancer cells indicated that the effective 5-FU concentration may be reduced to 2.5 times and up to 10 times, in case of sulforaphane and 5-FU in HT-29 and Caco-2 cells, respectively (Table 1). The reduction of effective drug levels is one of the main objectives in multiple-drug therapies. Hence, treatment efficacy increases, the therapeutic level of the drug decreases, and the toxicity of the drug, very often determining the success of an anticancer therapy, is minimized [20].

Literature data indicates that the occurrence of positive interactions increasing anticancer activity stemming from the administration of combined compounds is due to the appearance of similar mechanisms or enhancement of a particular effect [21]. Due to the fact that such an interaction leads to the stimulation of a cytostatic and/or cytotoxic effect, we further determined both effects for the most promising combinations (sulforaphane and 5-FU and alyssin and 5-FU) in colon cell lines.

### 2.2. Cell Cycle Distribution and Cell Viability

**Figure 4:**
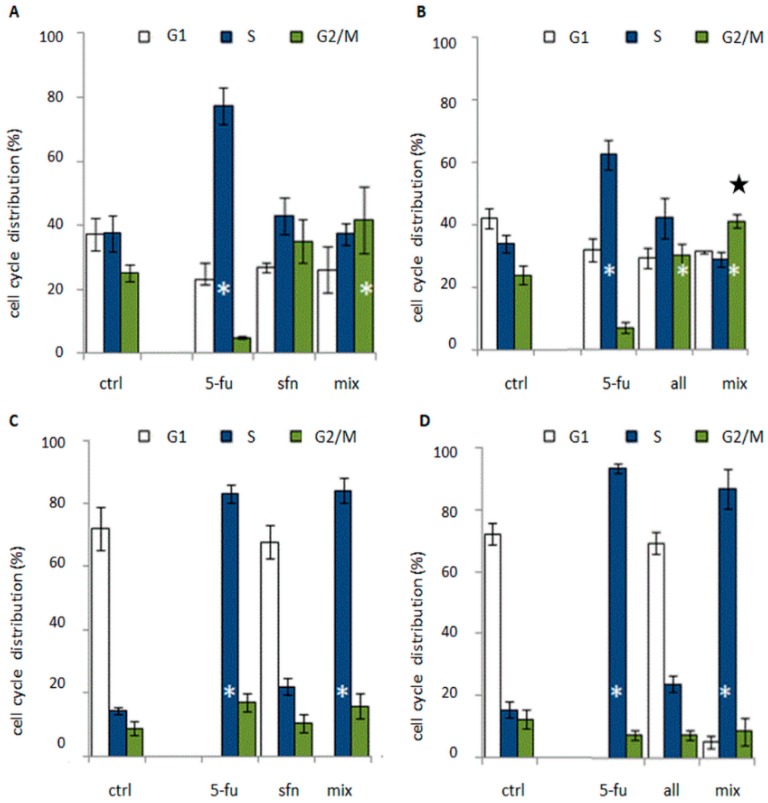
The changes in distribution of cell cycle phases in colon cell lines when compared to control after the administration of combination and compounds alone: 5-fluorouracil (5-FU) with: sulforaphane (sfn) in Caco-2 (**A**), and HT-29 (**C**) cell lines and alyssin in Caco-2 (**B**), and HT-29 (**D**) cell lines. Black star—the statistically significantly difference between the administration of combination and the singular administrations of ITC and 5-FU. White star—the statistically significantly difference between the administration of combination and the control cells, *p* < 0.05. The cell cycle distribution was determined by flow cytometry. The combination: cells were incubated with ITC for 24 h and then with 5-FU for 72 h. In singular administrations one component of the combination was used.

**Figure 5:**
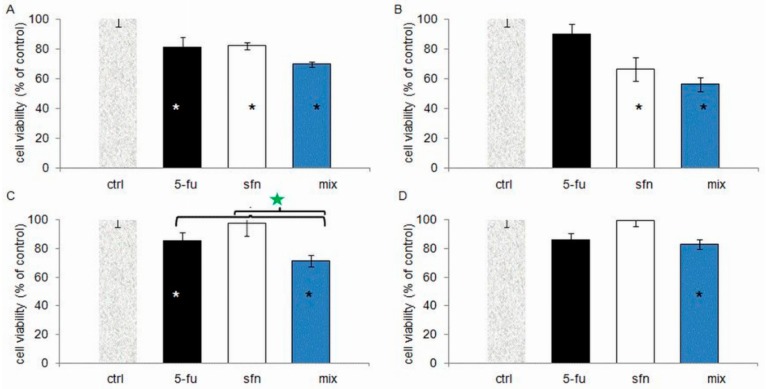
The changes of colon cancer cells viability after the administration of singular compounds and in combination: 5-fluorouracil (5-FU) with: sulforaphane (sfn) in Caco-2 (**A**), and HT-29 (**C**) cell lines and alyssin in Caco-2 (**B**), and HT-29 (**D**) cell lines. Green star—statistically significantly difference between the cell viability after the administration of combination and after administrations of ITC and 5-FU alone. * Statistically significantly difference from control. *p* < 0.05. The cell viability was determined by flow cytometry with the use of FDA/PI staining. The combination: cells were incubated with ITC for 24 h and then with 5-FU for 72 h. In singular administrations one component of the combination was used.

Cell cycle studies revealed that the combination of 5-FU and ITC blocked the cell cycle in the S or G2/M phase (Figure 4). The cytostatic activity profile, however, depended on the cell line. Reduced cell viability was observed in all tested combinations in comparison to the control group, which was correlated with an increase in the number of dead cells in the population (Figure 5). Typical of 5-FU anticancer activity, cell accumulation was observed in the S-phase after the 5-FU-ITC combined administration in HT-29 cell line of colon cancer. There was a statistically significant increase in the number of cells in this phase when compared to control cells, but at the level similar to singular administration of 5-FU. In both cases the effect was very pronounced as it was observed in 80 % of the cell population (Figure 4c,d).

In the case of HT-29 cells treated with the combination of 5-FU with alyssin, changes in the cell cycle act as a mechanism responsible for synergism. The mechanism of interaction between 5-FU and natural compounds, based only on affecting a cytostatic effect has been observed by a few research teams, e.g., the team of Espin, Li and Wu [22,23,24]. It is important to note that the team of S. Wu conducted their studies on an animal model and that they found the cytostatic effect sufficient to exert a decrease in the tumor size in comparison to the singular administration and the control mice [24]. All of the authors mentioned noticed a block in the S phase of the cell cycle, which is typical of 5-FU. By contrast, in Caco-2 cells administration of ITC combined with 5-FU induced accumulation of cells in G2/M, typical of ITC. The number of cells in this phase of the cell cycle was statistically significantly higher compared to control, and further enhancement of cytostatic effect was observed after the administration of alyssin combined with 5-FU with regard to singular alyssin administration (50 % more cells in this phase in comparison to singular administration of alyssin) (Figure 4b). This correlation was earlier observed in breast cancer cell line MDA-MB-231, where we proved the existence of synergism between 5-FU and sulforaphane [25].

In case of a combined administration of sulforaphane and 5-FU, in the HT-29 cell line, apart from a strong cell cycle block in S phase, additionally a decrease in cell viability occurred compared to singular administration of 5-FU (by about 20%) and sulforaphane (by about 40%, Figure 5). In this case we have also observed the highest decreases in cell growth (vide supra). Therefore, an intensification (synergism) of the anticancer effect took place after a combined administration of the compounds when compared to their administration separately. According to literature data, a strong cytostatic effect of a particular compound or an enhancement of this effect due to the combined administration are often accompanied by a cytotoxic effect. Such an integration of effects was observed, e.g., for the combination of paclitaxel and phenyl isothiocyanate [26]. This resulted from the fact that a cytostatic effect is caused by a checkpoint block in the interphase or mitosis resulting from DNA abnormalities. If the abnormalities are unrepairable, the cell dies [27]. Hence the last step of our study was to define the death pattern in HT-29 cells after administration of sulforaphane-5-FU combination.

### 2.3. Apoptosis Studies

Studies of apoptosis were conducted on the most promising cytotoxic combination (sulforaphane and 5-FU in HT-29 line). Apoptosis is considered the ultimate goal of an anticancer therapy. Since many researchers consider apoptosis resistance responsible for ineffective oncological treatment, new methods and combinations of compounds inducing cells’ susceptibility to this type of death are being sought [28]. Induction of apoptosis is in the focus of studies (in vitro and, more and more often, in vivo) on combinations of conventional drugs used in anticancer therapies with compounds of natural origin of a well-documented anticancer activity [29,30,31,32].

**Figure 6:**
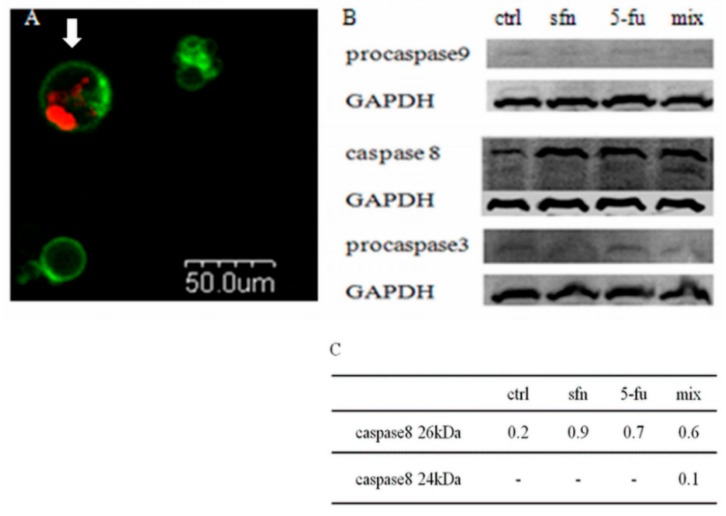
Detection of apoptosis. (**A**) Visualisation of apoptosis under confocal microscopy after the administration of 5-FU and sulforaphane, after 72 h incubation. Green—cell membrane stained with Annexin V-FITC. Red—a nuclei stained with PI. The arrow—a late apoptotic cell. (**B**) Changes in the levels of apoptosis-related proteins by western-blot after administration of the combination (mix) of sulforaphane (sfn) and 5-fluorouracil (5-FU) after 48 h incubation, and for the control cells (ctrl). Glyceraldehyde-3-phosphate dehydrogenase (GAPDH) was amplified as an internal control. (**C**) Bands of active caspase-8 forms were quantified by densitometric analysis and their intensity normalized with respect to GAPDH.

We have shown that the sulforaphane-5-FU combination induces a reduction in the number of the living cells and an increase in the number of the dead ones. Further examination showed that it was the result of apoptosis, as revealed by the Annexin V-FITC/PI test and microscopic observations (Figure 6a). Western blot studies were performed to confirm the results of the microscopic study. After administration of the combination a decrease in procaspase-3 level was observed. The study of the activation of apoptotic pathway proteins revealed two band for active caspase-8 forms and no changes in procaspase-9 level. These results indicate that observed by us apoptosis induced by sulforaphane-5-FU combination in HT-29 cells undergoes through a death receptor pathway (Figure 6b).

This mechanism of action resulting from sulforaphane and oxaliplatin was suggested by Kaminski et al. His team monitored the increase in the activity of oxaliplatin following an administration of sulforaphane in Caco-2 cell line through the induction of receptor mediated apoptosis [9]. The mentioned mechanism, however, is not typical for sulforaphane and sulforaphane-drugs interaction. Literature data indicates that sulforaphane induces apoptosis in combination with cytostatics (gemcitabine) by inhibiting the expression of antiapoptotic factors or mainly via mitochondrial pathway (with etoposide or adriamycin) [33,34]. Most often sulforaphane is recognized as an inhibitor of mitochondrial respiration and an inducer of apoptosis via intrinsic, mitochondrial pathway. Sulforaphane has also been shown to increase ROS level and activate AMPK pathways which led to mitochondrial apoptosis in gastric cancer cells [35]. Sulforaphane was also shown to induce apoptosis in pancreatic cell line through an inhibition of PI3K/AKT pathway [36]. In breast cancer cell line, sulforaphane promoted apoptosis via diminishing AKT signaling and at the same concentrations induced energy stress (reduced ATP pools) that caused AMPK activation and autophagy [37]. The results of our studies constitute hence a prospective proposal for the 5-FU and ITC mechanism of anticancer action in colon cancer cells.

## 3. Materials and Methods

### 3.1. Cells and Reagents

Colon cancer cell lines (Caco-2 and HT-29) and prostate cancer cell lines (LNCaP and PC-3) were obtained from the American Type Culture Collection (ATCC, Manassas, VA, USA). Colon cancers cells were grown in MEM (Cytogen, GmbH Bienenweg, Berlin, Germany). Prostate cancers cells were grown in RPMI (Cytogen). Mediums were supplemented with 20% (Caco-2) or 5% (Ht-29, PC-3, LNCaP) fetal bovine serum (Gibco, Grand Island, NY, USA), 1% antibiotics solution (10,000 U/mL penicillin and 10 mg/mL streptomycin, 25 µg/mL amphotericin B (Sigma Aldrich, St. Louis, MO, USA) and 1% nonessential amino acids (Sigma Aldrich). Sulforaphane and alyssin were synthesized as described previously by Schmidt and Karrer [38]. 5-FU was obtained from Sigma Aldrich.

### 3.2. Cell Growth Assay

Increasing concentrations of 5-FU and ITC were added to cells in the logarithmic phase of growth, next the incubation lasted 24 or 72 h. The cell growth was evaluated using the MTT (3,-4,5 dimethylthiazol-2,5 diphenyl tetrazolium bromide) assay. At the end of the incubation, the medium was removed and the 0.25 mg/mL MTT (Sigma Aldrich) was added. The absorbance of formazan was measured at a wavelength of 570 nm with background subtraction at 690 nm. The microplate scanning spectrophotometer (PowerWave X, BioTek, Winooski, VT, USA) was used for the measurements.

### 3.3. Quantitative Analysis of Interactions

To investigate the combined effect of ITC and 5-FU the Chou and Talalay method was used [17]. In the combination treatment studies, the tested substances were added in concentrations which were approximately their IC_50_ multiplicity. Sequential scheme of cell treatment was applied in case of each ITC and each cell line. The sequential scheme involves a 24-h pretreatment with ITC followed by treatment with 5-fluorouracil for 72 h. In addition, in the same experiments, the cells were incubated with each of the substances alone at concentrations corresponding to the concentrations used in the combined treatment. The effect was estimated with the MTT assay. To define the type of effect the Combination Index (CI) was calculated. The value of Combination Index (CI). CI > 1, CI < 1 and CI = 1 indicate antagonism, synergism and additive effect respectively. CI and DRI was calculated using CompuSyn software (ComboSyn, Paramus, NJ, USA).

### 3.4. Mechanism Investigation

The mechanism of interactions was investigated at the value of the percentage (fraction) of cells whose growth was affected (fa). 0.75. Values of the tested concentrations causing fa 0.75, were calculated using the CompuSyn software (ComboSyn). The assessement included: Sequential treatments and wand in addition, administartion of substances alone at concentrations corresponding to the concentrations used in combinations.

#### 3.4.1. Cell Cycle Analysis

The cytostatic effect of 5-FU treatment, ITC treatment and their combination administration were tested by the analysis of cell cycle distribution using the flow cytometry. After incubation the cells were trypsinized and rinsed with PBS and suspended in 75% ice cold ethanol. Each sample was stained with staining solution containing 50 µg/mL PI (Sigma Aldrich), 100 µg/mL RNase (Sigma Aldrich) and 0.1% Triton X-100 (Sigma Aldrich). The test determined the content of DNA, and according to the results, the phase of the cell cycle was defined. The cell cycle distribution was examined with FACS Calibur flow cytometer (BD Biosciences, San Jose CA, USA), and the CellQuest software (BD Biosciences). The cell cycle was analyzed with the Multi Cycle Analysis^TM^ Software (Phoenix Flow Systems, San Diego, CA, USA).

#### 3.4.2. Cytotoxicity Assay

The cytotoxic effect of 5-FU administration, ITC administration and their combination administration was examined by calculating the number of living and dead cells. Fluorescein diacetate and propidium iodide (PI) were used to stain live and dead cells, respectively). Cells were trypsinized and rinsed with PBS. Each 100 μL sample in PBS was treated with 10 μL of 6.25 g/mL fluorescein diacetate (Sigma-Aldrich) and 10 μL of 50 μg/mL PI (Sigma-Aldrich). After incubation on ice, 0.5 mL PBS was added and the sample was analyzed with the FACS Calibur flow cytometer. The cell viability was calculated using WIN MDI WinMDI 2.9 software (Becton-Dickinson).

#### 3.4.3. Microscopic Identification of Apoptosis

For the purposes of microscopy analyses the cells were stained with the FITC Annexin V Apoptosis Detection Kit I (BD Biosciences Company, San Jose, CA, USA) FITC Annexin V (5 μL per 1 mL buffer) and PI (5 μL per 1 mL buffer) fluorescence was excited with 488 nm and PI 543 nm lasers, respectively. The FITC and PI fluorescence were collected with help of a confocal microscope (Olympus, Shinjuk, Tokyo, Japan) using 520 nm and 600 nm filters respectively. There were distinguished: living cells (AnnV−/PI−), early apoptotic cells (AnnV+/PI−) and necrotic or late apoptotic cells (AnnV+/PI+).

#### 3.4.4. Western Blot Assay

Levels of proteins engaged in the apoptotic process (caspase-8, procaspase-9, procaspase-3), were determined by western blot anaysis. A sample was separated with SDS-PAGE and transferred to a PVDF membrane (Amersham, GE Healthcare Life Sciences, Freiburg, Germany) using a vertical electrophoresis apparatus Mini-PROTEAN ^®^ 3 Cell (BioRad, Hercules, CA, USA.). Proteins were immunoblotted with the primary monoclonal antibodies: anti- aspase-8, anti-glyceraldehyde-3-phosphate dehydrogenase (GAPDH) (Thermo Fisher Scientific, Waltham, MA, USA), anticaspase 3 and 9 (Cell Signaling, Leiden, The Netherlands). GAPDH was amplified as an internal control. Protein bands were visualized with the Bio Imaging System (DNR Lumi BIS, Jerusalem, Israel), using the fluorescent method of the Western Dot Kit (Thermo Fisher Scientific). Protein bands were characterized using ImagineR (Sun Microsystems, Santa Clara, CA, USA) analysis software.

### 3.5. Statistical Analysis

Statistical analysis was carried out using one-way analysis of variance (ANOVA) followed by a post-hoc Dunnett’s test, for separate comparisons with the untreated control and the post-hoc Tukey’s test was used to compare pairs of group means (*p* < 0.05). The statistical analysis was performed with GraphPad Prism 5 (GraphPad Software, Inc., La Jolla, CA, USA).

## 4. Conclusions

The aim of this paper was to determine the impact of the sulphur-containing phytochemicals: sulforaphane and its natural analog alyssin on the anticancer activity of 5-FU. The results of the study indicated that both ITC increased the anticancer activity of 5-FU. The tested combinations of 5-FU with ITC were the most effective in the HT-29 and Caco-2 colon cancer lines. The study revealed synergistic interactions between combination components, which allowed for a considerable reduction in the 5-FU effective concentration and more effective cancer cell growth inhibition. The block of cell cycle in S phase in HT-29 cells and G2/M in Caco-2 cells and an increase in dead/living cells ratio were observed.

The most beneficial anticancer properties have been shown for the combination of sulfopharane with 5-FU in HT-29 colon cancer cell lines. The most effective cell growth inhibition was the effect of a strong cell cycle arrest in the S phase and enhanced cytotoxic effect of combination in comparison to a single compound action. The study also showed that sulforaphane-5-FU combination induced death of HT-29 colon cells through the receptor—mediated apoptosis. The elongation of the sulforaphane carbon chain (as in alyssin) decreased the molecule’s ability to potentiate the anticancer effect of 5-FU and also changed the mechanism of the combination action. In the case of alyssin an intensification of cell cycle inhibition was observed after combined treatment.

In conclusion, the results presented by us shed new light on possible molecular targets and the intracellular action of 5-FU combined with sulforaphane and its analog in colon cancer cells. Because of the lack of a completely safe and effective treatment for colon cancer, which is one of the leading causes of deaths around the world, developing a new strategy based on 5-FU and ITC may have an impact on new, more effective treatments of the cancer. Bearing this in mind, we are going to continue our studies.

## Figures and Tables

**Table 1:** DRI values of 5-FU in colon cancer lines.

Cell Line	DRI of 5-FU	
	Sulforaphane	Alyssin
**Caco-2**	9.7 ± 0.9	6.3 ± 1.5
**HT-29**	2.6 ± 0.5	2.2 ± 0.4

## Abbreviations

5-FU	5-Fluorouracil
CI	Combination Index
DRI	Dose Reduction Index
PI	Propidium iodide
ITC	Isothiocyanates
MTT	3,-4,5 Dimethylthiazol-2,5 diphenyltetrazolium bromide
